# A Blind Man Leads a Blind Man? Personalised Nutrition-Related Attitudes, Knowledge and Behaviours of Fitness Trainers in Hungary

**DOI:** 10.3390/nu12030663

**Published:** 2020-02-29

**Authors:** Anna Kiss, Laura Pfeiffer, József Popp, Judit Oláh, Zoltán Lakner

**Affiliations:** 1 Faculty of Food Science, Szent István University, 1118 Budapest, Hungary; kiss.anna891@gmail.com (A.K.); laura.pfeiffer23@gmail.com (L.P.); 2 WSB University, 41-300 Dąbrowa Górnicza, Poland; poppjozsef55@gmail.com; 3 Department of Food Economics, Faculty of Food Science, Szent István University, 2100 Gödöllő, Hungary; lakner.zoltan@etk.szie.hu

**Keywords:** coaches, dietary counselling, gym, focus group, personalised nutrition, structural equation modelling

## Abstract

It is well-documented that fitness trainers could play an important role in the nutrition-related behaviour of their clients based on their personalised nutrition-related counselling activities, but there are considerable concerns all over the world about the level of their knowledge to become nutritional coaches. In the framework of the current study based on qualitative (focus-group interviews) and quantitative (questionnaire and analysis of responses by multivariable methods, as well as structural equation modelling) methods, it has been proven that (1) theoretically, both the trainers and the dietitians acknowledge the importance of cooperation in the optimisation of coaching efficiency and advisory work due to some “professional jealousness” and differences in professional background, as well as in culture, so it is hard to find a common platform for cooperation, especially in market segments characterised by relative low levels of purchasing power; (2) due to lack of regulation, there is a high heterogeneity of professional competences of trainers in general and their nutritional competences, in particular; (3) the majority of trainers do not have an objective picture on his/her effective nutritional knowledge, and they often offer a much wider scope of services (e.g., nutritional counselling for clients with chronic diseases) which are well beyond their professional knowledge and (4) the dietary guidelines have not become an integral part of professional knowledge, even at the level of specialists. To improve the current—in some cases, dangerous—situation, the following steps should be taken: (1) enhancement of the level of professional qualification of future trainers, integrating the practice-oriented approaches and emphasising the role of teamwork by simulation-based practices; (2) highlighting in a clear way the professional and ethical boundaries of the activities of trainers and (3) working out an efficient incentive system for the continuous professional development of trainers.

## 1. Introduction

There has been a worldwide increasing tendency in the attendance of fitness clubs (Thompson) [1] with the aim to begin a weight-loss journey (Rapport et al.) [2]; therefore, the number of gyms and personal trainers is collaterally growing as well. A gym is an ideal place for health improvement (Damásio et al. [3] and Dabija et al. [4]), where, in addition to exercising, conversations about nutrition also play a role of high priority, and, for that reason, fitness enthusiasts look for nutrition advice (Howley and Franks) [5], since this is a favourable possibility to establish and fix optimal nutritional behaviours (Malek et al.) [6], as well as dispel nutrition myths that are born and spread due to providing incorrect information [7].

Personal trainers are well-placed to practice the promotion of physical activity and to provide basic nutrition care all at once, because those trying to enhance their exercise adherence are very likely to ask for other health-related advice as well, such as for nutrition advice. In this sense, nutrition care includes all practices that are focused on transforming/changing the client’s dietary habits [8].

The lack of nutritional knowledge has been well-known and documented in the literature for more than two decades. Stacey et al. [9] published a review article on this topic. Some important primary research examples are summarised in Table 1. Obviously, the gap between the nutritional knowledge level and the demand can be considered as a global problem of the fitness industry.

A great striking example for that is the consumption of dietary supplements among recreational athletes, as well as the communication that goes around this subject between personal trainers and their clients. The prevalence of dietary supplementation among sports enthusiasts is 30–70%. As far as European countries are concerned, e.g., one-third of Italian recreational athletes sport lovers took dietary supplements based on the recommendation of their trainer; 18% of them were influenced by information found on the Internet, whereas 14% of them took some based upon their physician’s advice. The consumers were not aware of either the effects or the side effects of the supplements, and they followed their trainer’s orders passively. The authors believe that accurate guidance is especially important among gym-goers. Furthermore, the promotion of updated education for fitness instructors is also essential [19]. The same pattern of dietary supplement consumption has been noted among Spanish fitness enthusiasts. Out of 415 participants, 28% used protein powders regularly; of whom, 25% had at least one shake a day (57 g per day on average). Even though there was a nutrition specialist working in all of the fitness clubs participating in the survey, 70% of the consumers buy these dietary supplements based on a friend’s or a trainer’s recommendation [20]. Similar results can be seen in the case of developing countries. According to Saeedi et al. [21], in Teheran, 44.6% of those who go to a gym regularly find that it is crucial to be advised about dietary supplements, and their primary source of information was either their physician or their personal trainer [22].

The nutrition-related education in Hungarian fitness centres is a question of specific importance, because (1) obesity is a considerable problem for the Hungarian population, and the Hungarian prevalence of obesity is one of the highest in the world (Lukács et al.) [23]; (2) the fitness centres are often visited by young males with relatively low levels of education, and that is why this is an ideal place to achieve such population segments which are hardly achievable in other ways for health-related communication and (3) the Hungarian fitness industry is increasing intensively. This tendency is fuelled by a favourable economic climate (relatively rapid increasing of purchasing power) and liberal regulatory framework. Under these conditions, the quality of the service of gyms are rather heterogeneous. A consequence of the liberal regulatory policy is the heterogeneity of the qualification of trainers. Mushrooming of courses for trainers from two-week-long courses to high-level ones can be experienced. There are about a thousand personal trainers attending various personal trainer courses in Hungary every year (Bartha and Bába) [24], and more and more of them offer nutrition advice; however, there is no information regarding these trainers’ practises of nutrition care.

**Figure 1:**
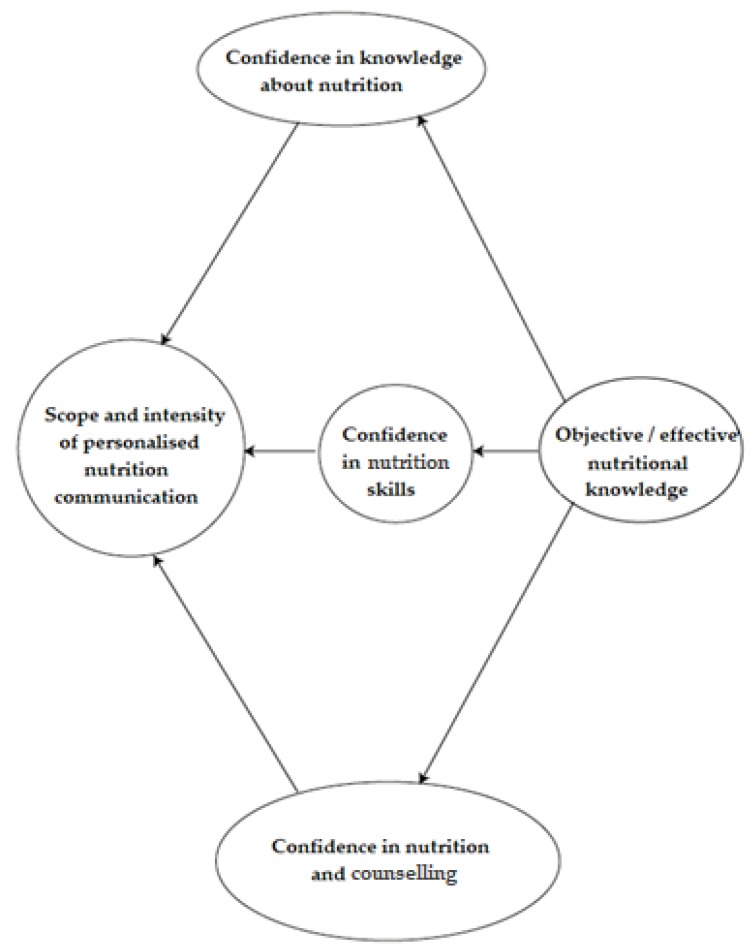
Visualisation of conceptual framework outlined in the H_3_ and H_4_ hypotheses.

This research analyses the current situation of nutritional advice practice in Hungarian fitness centres. Based on three sources: (1) a wide range of literature survey, (2) preliminary deep interviews (five face-to-face deep interviews with fitness trainers) and (3) the personal experiences of the authors: one of them has been working for four years as a part-time fitness instructor and another one as a part-time advisor for a company offering nutritional supplements for fitness centres and trainers. The remaining authors are active gym-goers. That is why our preliminary collection of information was based not only on classic research methodology but also on personal experiences, integrating the “live-in” approach of postmodern research (Holmes) [25]. Based on this preliminary information collection, five hypotheses have been developed:

- H_1_: There are considerable differences in the qualification levels of trainers. This can be explained by the fact that, currently, regulation of the trainer qualification system is rather liberal/loose;
- H_2_: The differences in the qualification of trainers are well-reflected in their self-reported level of confidence in nutrition-related knowledge and skills;
- H_3_: The intensity of nutrition-related communication (diversity of communication channels, content, scope and target groups) is determined by trainers’ self-perceived knowledge level;
- H_4_: There is a strong correlation between the self-perceived knowledge and the objective knowledge level. Figure 1 shows the visualisation of the H_3_ and H_4_ hypotheses.
- H_5_: The dietary guidelines are well-known for trainers because (a) these materials have been an integral part of each level of sport-related education, and (b) the first version of these guidelines were developed more than forty years ago, and one generation at a time should be enough to be filtered into the public consciousness.

Novelty of the current research is threefold:

1. Joint qualitative, as well as quantitative, research methods are used; this research is based on two qualitative-focus group interviews: one with trainers and another one with dietitians. By this method, a lot of pieces of information can be collected on such problems that are hard to evaluate by traditional “paper and pencil” methods.
2. Analysis of the attitude of trainers based on a complex questionnaire integrating two elements: (1) general characteristics of fitness trainers, their qualification and scope of nutrition-related communication; and (2) survey of their attitudes and self-evaluation of nutrition-related knowledge, skills, communication and counselling behaviour, as well as objective knowledge.
3. The mutual relationship between different factors have been analysed by methods of modern structural equation analysis.

## 2. Research Methodology

### Procedure

**Figure 2:**
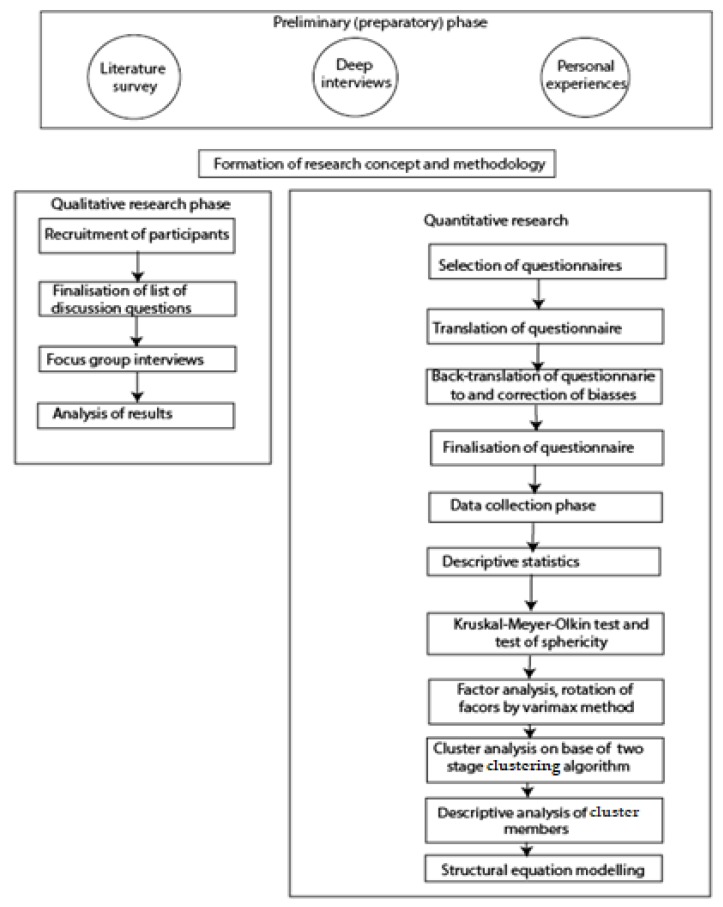
Flowchart of the investigations. The overwhelming majority of trainers and dietitians are active in community media, because this is the main channel of promotion by keeping regular contact with their clients and peers. This vibrant ecosystem of trainers has been used to contact them and recruit the participants of the survey by using the direct-contact and the snowball methods.

The phenomenon under investigation is a highly complex issue going far beyond the simple measurement of effective or tacit knowledge and attitudes by applying a combination of quantitative and qualitative methods. The flowchart of the investigation is presented in Figure 2.

In the first phase, two focus-group interviews were organised. The participants of the focus group interview with the trainers were recruited in Budapest based on personal acquaintances with one of the authors (LP). There was no day-to-day contact with each other among the trainers, but a professional acquaintance could not be excluded. Theoretically, the way of recruitment could lead to a bias, but during the analysis of the community media (blogosphere, Instagram, Facebook and Twitter profiles), it became evident that the world of Budapest fitness trainers is a very close-knit community. This statement was proven by a simple network analysis: out of 20 randomly chosen fitness trainers, any other trainer could be reached in a maximum of three steps. In the process of recruitment, we purposefully tried to involve different segments of trainers. So, it can be supposed that the results of the focus-group interviews reflect properly the general structure of fitness trainers in Hungary; however, willingness to participate in the study was relatively low. There were eight participants in the trainer-focus group. Five participants had at least a BS degree—three of them had high school qualifications. The average age of participants was 32 years, and the length of the average practice as trainers was 4.8 years. Five of them were males and three of them females. All of them were personal trainers offering nutrition-related advice to their clients. In a focus-group interview with dietitians, the number of participants was nine. The sample was dominated by women, and just one participant was male. All of the participants had at least a BS degree in dietetics, and all had, to some extent, contact with fitness: all were gym-goers, and three participants were working as part-time dietetic advisors in a fitness centre.

The questionnaire was completed by 264 fitness instructors, of which, 164 (62.2%) were females and 100 (37.8%) males. The age of the participants varied between 19 and 51 years, and the mean age was 30 years (M = 30.96; SD = 7.10). As far as the place of residence was concerned, 60.4% of the respondents lived in the capital city and 6.7% lived in a village at the time of the survey. 

The basic instrument of the survey was the Nutcomp tool with a general purpose. This was a validated questionnaire to measure the self-perceived competence of primary health professionals in providing nutrition-related information. This questionnaire is widely applied in international practices [26]. The original questionnaire has been modified in two points: (1) the references of the Australian guidelines have been changed to analogous Hungarian documents. The latest dietary guideline for the healthy adult population in Hungary is the SMART PLATE^®^, published by the Hungarian Dietetic Association. SMART PLATE^®^ is based on the recommendations of the previous Dietary Guidelines to the Adult Population in Hungary. It is a food-based dietary guideline and provides dietary recommendations in connection with the main food groups: fruit and vegetables, grains and cereals, milk and dairy, meat, fish and eggs. The guidelines also recommend moderate consumption of fat, sugar and salt, as well as support to create a hydration strategy, healthy portion sizes and recipes [27]; and (2) item no. 10 in chapter “Confidence in nutrition skills” was discarded, because based on the experience of the preliminary question-testing phase, numerous misunderstandings happened concerning the concept “peer-reviewed” and “evidence-based”. 

The effective/measurable food and nutrition-related knowledge of trainers was measured based on the shortened version of the Nutrition for Sport Knowledge Questionnaire (NSKQ) validated and used widely to survey the nutrition-related knowledge of athletes [28,29].

All necessary materials, including the detailed research plan, has been presented to the Research Ethics Council of the Doctoral School of Szent Istvan University (Gödöllő, Hungary). This council approved it. All participants of the focus-group interviews filled out an “informed consent” declaration. The questionnaires, filled out by participants, have been evaluated by an independent legal specialist of the Hungarian Academy of Sciences, taking into consideration the general data protection regulation (GDPR) requirements of the EU [26].

## 3. Results

### 3.1. Results of Focus-Group Interviews

#### 3.1.1. Results of Focus-Group Interviews with Trainers

The trainers agreed that nutrition is very important for the clients, both in terms of health preservation and in terms of having an appealing appearance. These are the main motivators for a gym-goer in seeking the help of a trainer. Trainers think nutrition and exercising are closely related and that nutrition care plays a main role in a trainer’s scope of activities, because training and nutrition are inseparable. As one of the participants has formulated:

“In efficiency of workout, 40% has the nutrition. If the client wants to lose weight, nutrition plays a role of 70%. I am no more, just a booster of these processes.”

As far as the interpretation of nutritional care is concerned, it varies significantly from suggestions and sample diets to personalised ones and to even changing macronutrient ratios drastically. Diets followed by the fitness instructors themselves correspond with their conviction in nutrition, with slight differences in their diets being stricter, including more restrictions. One typical statement:

“The client journals her diet for a week then I put together a diet for her, but not a drastic one yet so that her body can adjust. Then we cut back on carbs automatically and calculate the macronutrients and she has to follow a diet according to that.”

In the framework of focus-group interviews, it became clear that fitness instructors use neither national nor international nutritional guidelines during nutrition care. Neither of them feels that they go against these guidelines, but they think these national guidelines made for the adult population are not relevant to their clients.

“In general, there is something useful in each piece of nutrition advice. I like to get to know them and then decide how much sense I find in them. There are a lot of things in dietitians’ suggestions that I do not agree with, such as consuming complex carbs.”

All of the instructors participating in the survey declared that they would find it profitable if professional nutrition organisations made their studies and articles available for trainers. All of them stated that cooperation between trainers and dietitians would be important, but they expressed their concerns about purchasing the power needed to buy the augmented services: “It sounds very nice to offer complex solutions to the clients, but in our district there is not enough purchasing power for this. How could a simple gypsy guy pay for extra dietitian?” or “I have attended a course of trainers to earn money. If here comes a dietitians why had I learned? I could throw away half of my qualification!”

#### 3.1.2. Focus-Group Interview Completed by Dietitians

In the opinion of the dietitians, a gym can be an ideal place to educate people about healthy diets, because those who attend a gym are much more willing to actually change their lifestyle and dietary habits than those who go to see a dietitian. The dietitians who completed the interview agreed that it would be relevant to initiate building a cooperation based on the relationship between dietitians and personal trainers and to refer to each other during their job. “It could be a good direction, useful as well, but how it would work out depends on the implementation.” In some cases, preliminary preparatory activities had been reported, too: “Practice communities are being planned. Including physicians and therapists in primary care, it could as well make a common practice where training and physical activity also get a role.” Some respondents highlighted the role of differences in qualification and background. As one of them stated: “If you are a professional dietitian, you have to accomplish at least a college, lay down serious examinations on anatomy, physiology, etc... It is rather hard to me to accept a trainer, with a super-size ego and with a two-weeks long course as a peer.” or “It is often uneasy to me to cooperate which such persons, who are not able to discriminate between scientific facts and public hype. I do not want to waste my time and energy to dismiss obvious lies and myth, which are well-accepted, just because they are present in the community media.”

As a summary, it can be stated that, in theory, the trainers and the dietitians agreed on the importance of cooperation, but in practice, there has been a certain level of “professional jealousy” from the side of the trainers, most probably due to limited purchasing power for the service of fitness.

### 3.2. Results of the Quantitative Survey

Results of the survey has proven a high level of heterogeneity in professional preparedness of trainers. Just 18% of respondents have accomplished at least a BS/BA qualification (mainly in the field of sport and recreational sciences), 65% have a qualification as fitness trainers in the framework of the National Qualification Register (post-secondary education); the remaining have just a certification of attendance of some short-term course. The majority of the respondents (57.9%) graduated from higher education, while 42.2% graduated from secondary education.

**Figure 3:**
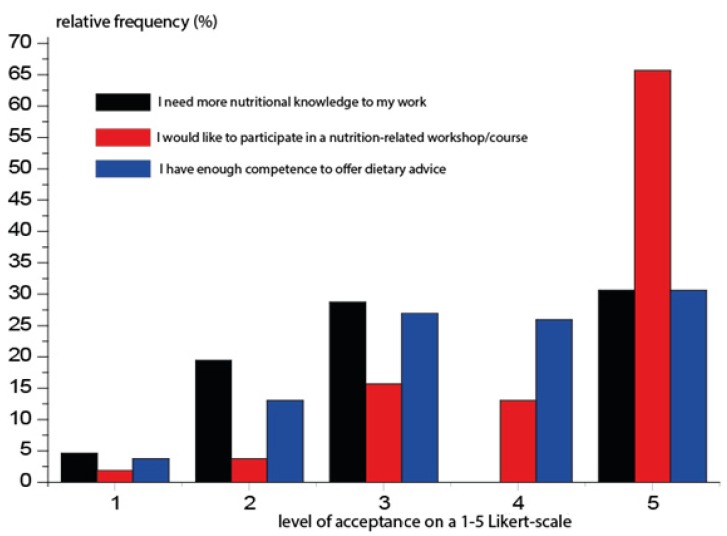
Attitudes of trainers to nutrition-related knowledge.

In line with focus-group interviews, the attitude of trainers to enhancement of their nutrition-related knowledge has been positive (Figure 3).

The first series of statements concern the attitudes towards nutrition care. As it has been assumed based on focus-group interviews, the majority of respondents attached a high importance to nutrition in fitness training (Table 2). This fact is mirrored not just by high mean values but also in low values of standard deviation. The statement concerning the nutrition of clients with chronic diseases has divided the respondents, as the majority of them do not offer specific counselling services for this target group, which can be considered as normal.

In first phase of the investigation, we have determined the descriptive features of self-reported knowledge levels about nutrition and chronic diseases, as well as the ability to understand and/or manage nutrition-related activities. Results are summarised in Table 3. Analysis of results has offered a rather bizarre result: the respondents have been rather self-confident on nutrition care, even in cases of the formation of recommendations to individuals with chronic diseases, as well as in determining appropriate food and nutrition goals. At the same time, the self-reported knowledge of the effect of foods and nutrients on the body system has been much lower.

The inherent structure of the database has been analysed by factor analysis. Rotation of factors has been done by the varimax method. Results of the factor analysis yielded three factors explaining 70.6% of the total variance. Interestingly, the knowledge and application of dietary guidelines have been in separate factors (Table 4).

The first factor comprised mainly the self-perceived confidence; the second one, the evaluation of self-perceived knowledge. The interpretation of individual’s biological data is included in the second factor. This fact highlights that this document has not become an integral part of the professional culture; however, it was published more than forty years ago [30].

The individuals based on their factor scores have been clustered (Table 5). Two clusters could be separated: the clusters of self-confident and lesser self-confident responders. This in itself could be considered as an evident fact, but it is very curious and characteristic to the current situation that the members of both groups considered themselves able to “recommend changes in food choices for an individual with chronic disease” and to provide nutrition care which results in improvements in the food that an individual usually eats.

The respondents were highly confident in the application of different communication and counselling skills (Table 6). Based on a self-evaluation of communication and counselling skills, just one factor could be separated. This further supports the statement formulated in the previous paragraph: there is a considerable gap even on the level of self-reported knowledge and the perceived competence in trainers. Put another way: the trainers, who evaluate their knowledge and competences as relatively low, consider themselves capable to offer advice.

**Figure 4:**
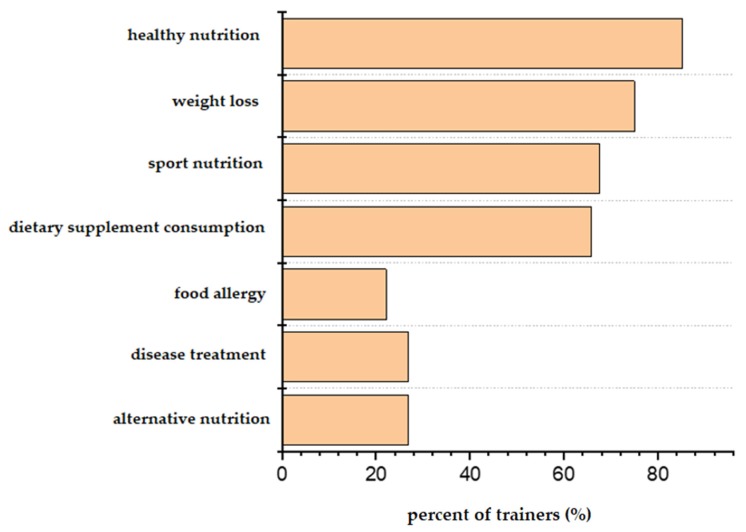
Content of nutrition-related advisory and counselling activity of trainers (percent ratio of trainers mentioning the content as a part of their counselling activity).

**Figure 5:**
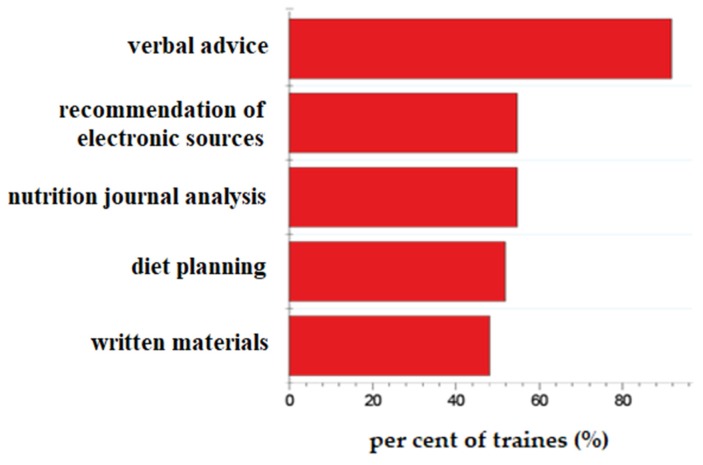
Form of nutrition related advisory activity.

In the next phase, we have tested the H_3_ and H_4_ hypotheses. Obviously, the trainers offer a wide-range of nutrition-related counselling activity (Figure 4 and Figure 5). The majority of the trainers give advice to their clients in the fields of general healthy nutrition, weight loss and sport nutrition. More than 80% of the trainers provide verbal advice, but they also recommend electronic sources of nutritional information and plan individualised diets.

The intensity of the nutrition-related activity of trainers has been evaluated based on a 1–10 scale depending on a portfolio of their nutrition-counselling activities and the channels of communication. The shelf-reported contents of communication (applying now closed-ended questions) were as follows:

- Healthy nutrition
- Sport nutrition
- Weight loss
- Consumption of dietary supplements
- Food allergy

Forms/channels of communication:

- Oral counselling
- Nutrition planning
- Food diary analysis
- Written materials
- Electronic materials (blogs and websites)

Each content item and form of communication got one point. In this way, a maximum of ten point could be achieved. If one trainer communicated on each topic by using each a channel, he or she got ten points. The background variables have been approximated by directly measurable variables according to the scales of the Nutcomp and NSKQ questionnaires. In the first phase of the investigation, we tested the relationship between perceived knowledge, nutrition and communication skills and the communication intensity. 

Results of the model-fitting are summarised in Table 7. It can be noted that the model fit extremely well, highlighting that the communication intensity is influenced in a significant way by perceived self-confidence in nutrition and communication.

In the next phase, we tried to fit a structural equation to the model, including the effective knowledge on nutrition measured by the NSKQ questionnaire. The results of this test were, on average, 75% with a considerable standard deviation (22%). In this phase, we were not able to determine any significant stochastic relationship between the effective test results and self-reported values.

## 4. Discussion and Conclusions

Results of the current study are in line with the main conclusions of similar studies. Results correlate with the ones found in the research made by Weissman et al. [12], in which 91.5% of the trainers who participated in the survey admitted to providing nutrition care to their clients. Results show that over 70% of trainers offer nutrition advise. Based on the results, two-thirds of the instructors are completely open to professional development and, in order to do so, they are willing to attend gratuitous workshops or courses. In the United States, 93% of fitness instructors are willing to attend free courses [12].

Results support the rather contradictory picture on nutrition-related levels of preparedness of trainers but, based on a combination of different methods, the results of the research add some new insights to the problem. The focus-group interview supported the practical difficulties in the achievement of rapid success in this situation. These problems (e.g., the work of a dietetic specialist in a gym means another cost to clients) have gotten less attention in the literature; however, the article of Manore et al. [31] must be highlighted. We compared the results of the focus-group interviews with the results of the qualitative study published by Barnes et al. [14]. For Australian personal trainers, nutrition optimisation is a significant part of their scope of practice, as it is very aligned with their clients’ goals. Based on the results of our survey, it is also apparent that, according to the trainers’ opinions, nutrition and training are closely connected; nutrition care is a major part of their practice, as training and nutrition are indispensable. Therefore, the practice of nutrition care is one of the main determining parts of their scope of activities. The trainers are aware of the necessity of evidence-based nutrition care; they ranked academic literature as the most authoritative source of information in nutrition science. By contrast, based on the fitness instructors’ approaches, neither the Australian nor the Hungarian personal trainers considered national nutrition guidelines proper enough, and as a consequence of no other evidence-based guidelines available, they felt the need to offer something” different”. Instead of evidence-based nutrition care, they handed over-information to their clients based on their own conviction, as well as information coming from fellow trainers [14].

There are considerable differences in the level of self-evaluation of preparedness of the trainers. This is in line with the H_1_ hypothesis. This phenomenon can be explained by three factors: (1) the regulation is liberal, (2) fitness trainers became a fashionable profession with a high demand for different courses, offering some fitness trainer qualifications and (3) there is a fierce competition between different gyms, contributing to decreasing costs by hiring often low-quality or under-qualified trainers. The considerable differences in the level of self-evaluation of preparedness of the trainers supports the H2 hypothesis, but this is not reflected in their intensity of communication, so the H3 hypothesis is rejected. There is no significant correlation between self-reported knowledge and skills level, as well as objectively measured knowledge, thus H4 hypothesis is not supported. According to study results of Skopinceva [32], high-qualified coaches were less confident in their nutrition knowledge compared to trainers who hold certificates. Beliefs and attitudes of the trainers were statistically not significant.

The Hungarian national guidelines of healthy eating could serve as a minimal, mutual platform for the improvement of nutritional communication and counselling activities, but even these simple statements have not become a part of the professional culture, and so, the H_5_ hypothesis is disapproved. It should be mentioned that SMART PLATE^®^ is the only national guideline for the healthy adult population, and there is no other official platform where the trainers could find reliable information or educate him/herself regarding the nutrition aspects of chronic diseases.

Results of the quantitative analysis have proven a considerable difference between self-reported and effective nutritional knowledge of trainers. This is especially problematic in the case of nutritional counselling to persons with chronic diseases. A potential way of explaining this contradiction can be based on four pillars: (1) the level of qualification (and health culture) of the majority of trainers is too low to understand the importance of professionalism in nutrition counselling. This supposition is well deductible from the relatively low levels of education of the natural sciences in Hungarian educational system in general by Henard and Luca [33], and biology in particular [34]. The current forms of trainers’ educations are not sensible for future trainers due to the boundaries of their competence in nutritional counselling; (2) the adverse effect of community media. The analysis of the blogosphere, Facebook, Instagram and Twitter activity of trainers showed that practically each trainer had materials on “healthy” eating. These pieces of information have advocated some popular diet and eating patterns despite the lack of any scientific background. The misleading effect of community media is well-documented in academic literature [35,36]; (3) the high level of self-confidence of opinion leaders in community media (independently of the content of their message) can serve as a pattern for trainers in the development of the form and content of their communication strategy and tactics. This phenomenon is analysed by Weeks et al. [37]. There is an increasing, fierce competition between different gyms; therefore, the trainers try to enhance the number of their clients by offering a wide-range, complex service, even if their knowledge background is rather weak.

The solutions should be based on a complex, holistic approach, of which the most important elements are the following steps: (1) rigorous, national-wide regulation of gyms, taking into consideration the opinion of professional organisations, fitness specialists and specialists from different public health service agencies (e.g., Chief Medical Officer Office, National Food Safety Authority, etc.); (2) based on minimal the requirements of opening and running a gym, a self-regulatory quality control system should be introduced in the recreation centres integrating the nutrition-related services; (3) upgraded qualification systems of trainers are badly needed, emphasising the impact of nutrition on sport performance and health; (4) the education of trainers should help (a) to find scientific quality, peer-reviewed nutrition-related materials and (b) based on situational training and case studies to demonstrate the efficiency of teamwork integrating different disciplines; (5) besides the regulations, some ethical guidelines should be applied to orientate the trainers not going beyond their competences and (6) info-sheets and other well-organised materials should be placed in gyms to promote the importance of healthy nutrition and dispel the nutrition mythos.

The main limitations of the current study were the small number of participants in the qualitative phase of this research. However, results are similar to other significant qualitative studies in assessing the nutrition-related knowledge, attitudes and practices of fitness trainers.

## Figures and Tables

**Table 1:** Low level of nutritional knowledge is a general problem of the fitness industry—some examples from the literature.

Source	Year	Country and Method	Results
Kruseman et al. [10]	2008	Switzerland, questionnaire	Sixty percent of fitness instructors self-rated their nutritional knowledge as insufficient.
Torres-McGehee et al. [11]	2012	USA, questionnaire,	The level of effective knowledge of athletic trainers and strength and conditioning specialists is acceptable, but in the case of coaches, not.
Weissman et al. [12]	2013	USA, questionnaire	Forty-six US states prohibit the practice of dietetics and nutrition without a minimum education and training level. In spite of this, the trainers give advice and charge for it without adequate performance.
McKean et al. [13].	2015	Australia, online survey	Low levels of modern nutritional qualification; most important sources are health magazines. The nutritional advice of trainers should be limited to nonmedical nutritional information.
Barnes et al. [14]	2016	Australia, website analysis	A considerable part of the nutrition content of websites of gyms is at-risk and low quality, misrepresenting the roles and limitations of personal trainers.
Barnes et al. [8]	2016	Australia, questionnaire	The personal trainer’s confidence was relatively low in nutrition and high in communication and advancing.
Barnes et al. [15]	2017	Australia, qualitative research	There is a considerable gap between the level of education in the field of nutrition of trainers and the knowledge they need.
Feldvari et al. [16]	2018	Croatia, questionnaire	Low level of nutritional knowledge.
McKean et al. [17]	2019	Australia, online survey	Low level of nutritional knowledge of trainers; complex teams including dietitians should be organised.
Tanacković [18]	2019	Croatia, questionnaire	Preferred sources of information of trainers are Google and community media.

**Table 2:** Attitudes towards nutrition care, measured on a 1–5 Likert scale (1—absolutely not agree and 5—totally agree) in descending ordering, according to mean value.

Statement	Mean	Std. Deviation
If the topic arises, it is important that I encourage my patients/clients to eat healthy foods.	4.70	0.645
It is important that all individuals usually eat healthy foods, regardless of age, body weight and physical activity levels.	4.66	0.686
Encouraging my patients/clients to eat healthy foods is within my scope of practice.	4.61	0.708
Providing specific nutrition recommendations to my patients/clients that can assist with managing their chronic disease is within my scope of practice.	4.58	0.908
Encouraging my patients/clients to eat healthy foods is an effective use of my professional time.	4.55	0.741
It is important that I take every opportunity possible to encourage my patients/clients to eat healthy foods.	4.31	0.882
Providing specific nutrition recommendations to my patients/clients that can assist with managing their chronic diseases is an effective use of my professional time.	2.65	1.369

**Table 3:** Results of descriptive statistics on nutrition-related knowledge and skills, measured on a 1–5 Likert scale (1—absolutely not agree and 5—totally agree) in descending ordering, according to mean value.

Self-Reported Knowledge of/Competence/Ability to...	Mean	Std. Deviation
Provide nutrition care that results in improvements in the food that an individual usually eats.	4.30	0.87
Recommend changes in food choices for an individual with chronic disease.	4.24	0.96
Maintain clear and concise records regarding the nutrition-related assessment and advice you provide to individuals.	4.19	0.92
Determine appropriate food or nutrition goals for an individual with chronic disease.	4.14	0.91
Collect information on the food consumption of an individual.	4.11	0.90
Interpretation of anthropometric data.	3.98	0.85
Monitor and evaluate changes over time regarding the food an individual usually eats.	3.92	1.02
Impact of an individual’s body composition on the development of chronic disease.	3.59	0.90
Guidelines for the nutrition-related management of specific chronic diseases.	3.52	1.06
Effect of foods and nutrients on body systems.	3.49	0.94
Interpretation an individual’s biological data.	3.39	1.01
Influence of foods and nutrients on the development and management of chronic disease.	3.38	1.04
Formulate a meal plan for an individual with chronic disease.	3.34	1.20
Use the Hungarian Guide to Healthy Eating.	3.29	1.16
The Hungarian Guide to Healthy Eating.	3.18	1.20
Current academic evidence regarding nutrition and chronic disease.	2.64	1.11
Interactions between nutrients with medications.	2.32	1.11

Remark: To save space, the sentences are slightly shortened versions of the statements of the original questionnaire.

**Table 4:** Results of the factor analysis on nutrition-related knowledge and personalised nutrition-related skills.

Self-Reported Knowledge of/Competence/Ability to...	Component		
	1	2	3
Effect of foods and nutrients on body systems.		0.804	
Influence of foods and nutrients on the development and management of chronic disease.		0.852	
Impact of individual’s body composition on the development of chronic disease.		0.706	
The Hungarian Guide to Healthy Eating.			0.880
Guidelines for the nutrition-related management of specific chronic diseases.		0.662	
Interactions between nutrients with medications.		0.813	
Current academic evidence regarding nutrition and chronic disease.		0.715	
Interpretation of anthropometric data.	0.684		
Interpretation of an individual’s biological data.	0.433	0.649	
Collect information on the food consumption of an individual.	0.813		
Use the Hungarian Guide to Healthy Eating.			0.837
Determine appropriate food or nutrition goals for an individual with chronic disease.	0.803		
Formulate a meal plan for an individual with chronic disease.	0.520		
Recommend changes in food choices for an individual with chronic disease.	0.884		
Monitor and evaluate changes over time regarding the food an individual usually eats.	0.739		
Maintain clear and concise records regarding the nutrition-related assessment and advice you provide to individuals.	0.836		
Provide nutrition care that results in improvements in the food that an individual usually eats.	0.897		

Remark: To save space, the sentences are slightly shortened versions of the statements of the original questionnaire.

**Table 5:** Self-reported competence evaluation concerning nutrition-related issues, measured on a 1–5 Likert scale (1—absolutely not agree and 5—totally agree), significant differences at *p* = 5% and levels are indicated by *.

Self-Reported Knowledge of/Competence/Ability to...	Cluster Number of Case	
	1	2
	Mean	Mean
Effect of foods and nutrients on body systems *.	3.87	3.09
Influence of foods and nutrients on the development and management of chronic disease *.	3.82	2.92
Impact of an individual’s body composition on the development of chronic disease *.	3.93	3.25
The Hungarian Guide to Healthy Eating *.	3.95	2.38
Guidelines for the nutrition-related management of specific chronic diseases *.	3.98	3.04
Interactions between nutrients with medications *.	2.76	1.87
Current academic evidence regarding nutrition and chronic disease *.	3.27	1.98
Interpretation of anthropometric data *.	4.09	3.87
Interpretation an individual’s biological data *.	3.67	3.09
Collect information on the food consumption of an individual *.	4.29	3.92
Use the Hungarian Guide to Healthy Eating *.	3.87	2.68
Determine appropriate food or nutrition goals for an individual with chronic disease.	4.05	4.23
Formulate a meal plan for an individual with chronic disease *.	3.60	3.08
Recommend changes in food choices for an individual with chronic disease *.	4.25	4.23
Monitor and evaluate changes over time regarding the food an individual usually eats.	4.09	3.74
Maintain clear and concise records regarding the nutrition-related assessment and advice you provide to individuals.	4.31	4.08
Provide nutrition care that results in improvements in the food that an individual usually eats.	4.35	4.25

Remark: To save space, the sentences are slightly shortened versions of the statements of the original questionnaire.

**Table 6:** Self-reported confidence in communication and counselling about nutrition, measured on a 1–5 Likert scale (1—absolutely not agree and 5—totally agree) in descending ordering, according to mean value.

Confidence in Ability to...	Mean	Std. Deviation
Communicate with clients about food and nutrition using culturally appropriate language.	4.40	0.875
Maintain a nonjudgemental attitude in discussions with patients/clients about the food they eat.	4.38	0.904
Demonstrate genuine empathy to patients/clients about their food-related experiences and goals.	4.35	0.835
Clearly describe what patients/clients can expect from their discussions with you about food or nutrition.	4.33	0.843
Identify individuals who need additional support from other health professionals or services regarding the food they eat.	4.25	0.898
Consider how personal, social, cultural, psychological and economic factors may influence the foods that a patient/client eats.	4.24	0.936
Work with patients/clients to identify possible ways to improve the food they usually eat.	4.04	0.906
Communicate with other health professionals about the discussions you have had with patients/clients regarding food.	3.98	1.023
Check a patient’s/client’s understanding of the influence of food and nutrients on their health.	3.97	0.932

**Table 7:** Results of the structural equation model, without taking into consideration the effective/measurable nutrition-related knowledge (z_crit_ = 1.96 at *p* = 0.05). Model-fitting parameters: *p* = 0.9, goodness of fit index = 0.95, adjusted goodness of fit index = 0.91 and comparative fit index = 0.92 (NA—nonavailable due to computational restrictions).

Statement	Estimation	Standard Error	z Value
**Self-reported knowledge of/competence/ability to...**			
Effect of foods and nutrients on body systems.	1	0	NA
Influence of foods and nutrients on the development and management of chronic disease.	1.217	0.128	9.526
Impact of individual’s body composition on the development of chronic disease.	0.963	0.113	8.495
The Hungarian Guide to Healthy Eating.	0.633	0.162	3.895
Guidelines for the nutrition-related management of specific chronic diseases.	1.039	0.135	7.702
Interactions between nutrients with medications.	1.067	0.142	7.491
Current academic evidence regarding nutrition and chronic disease.	1.049	0.142	7.373
**Nutrition-related skills**			
Interpretation of anthropometric data.	1	0	NA
Interpretation of an individual’s biological data.	0.948	0.162	5.845
Collect information on the food consumption of an individual.	1.345	0.157	8.545
Use the Hungarian Guide to Healthy Eating.	1.003	0.187	5.358
Determine appropriate food or nutrition goals for an individual with chronic disease.	1.149	0.156	7.375
Formulate a meal plan for an individual with chronic disease.	1.156	0.194	5.971
Recommend changes in food choices for an individual with chronic disease.	1.311	0.147	8.902
Monitor and evaluate changes over time regarding the food an individual usually eats.	1.337	0.167	7.991
Maintain clear and concise records regarding the nutrition-related assessments and advice you provide to individuals.	1.299	0.148	8.767
Provide nutrition care that results in improvements in the food that an individual usually eats.	1.324	0.157	8.432
**Communication and counselling skills**			
Clearly describe what patients/clients can expect from their discussions with you about food or nutrition.	1	0	NA
Check a patient’s/client’s understanding of the influence of food and nutrients on their health.	1.045	0.118	8.883
Work with patients/clients to identify possible ways to improve the food they usually eat.	1.148	0.13	8.818
Demonstrate genuine empathy to patients/clients about their food-related experiences and goals.	1.046	0.128	8.188
Maintain a nonjudgemental attitude in discussions with patients/clients about the food they eat.	1.054	0.116	9.073
Communicate with clients about food and nutrition using culturally appropriate language.	1.141	0.126	9.071
Consider how personal, social, cultural, psychological and economic factors may influence the foods that a patient/client eats.	1.125	0.121	9.267
Identify individuals who need additional support from other health professionals or services regarding the food they eat.	1.142	0.131	8.721
Communicate with other health professionals about the discussions you have had with patients/clients regarding food.	1.103	0.125	8.793
Effect of communication intensity	0.655	0.151	4.345
Self-reported knowledge and ability level.	1	0	NA
Self-reported nutrition skills level.	1.258	0.263	4.776
Self-reported communication skills level.	1.289	0.261	4.937

## References

[1] Thompson W.R. (2017). Worldwide survey of fitness trends for 2018: The CREP edition. Acsm’s Health Fit. J..

[2] Rapport F., Hutchings H., Doel M.A., Wells B., Clement C., Mellalieu S., Shubin S., Brown D., Seah R., Wright S. (2018). How Are University Gyms Used by Staff and Students? A Mixed-Method Study Exploring Gym Use, Motivation, and Communication in Three UK Gyms. Societies.

[3] Damásio A., Campos F., Gomes R. (2016). Importance given to the reasons for sport participation and to the characteristics of a fitness service. Arena J. Phys. Act..

[4] Dabija D.C., Abrudan I.N., Postelnicu C., Dunay A. (2015). Competitive strategies of fitness gyms in international business environment. empirical findings through observation. Proceedings of the 5th International Conference on Management (ICoM 2015). Management, Leadership and Strategy for SME’s Competitiveness.

[5] Howley E.T., Franks B.D. (1986). Health/Fitness Instructor’s Handbook.

[6] Malek M.H., Nalbone D.P., Berger D.E., Coburn J.W. (2002). Importance of health science education for personal fitness trainers. J. Strength Cond. Res..

[7] Rosenbloom C. (2008). Practical Considerations in Applying Theory: How Can We Narrow the Gap between Sports Science and Professional Practice in Sports Nutrition? A Commentary. Int. J. Sports Sci. Coach..

[8] Barnes K., Desbrow B., Ball L. (2016). Personal trainers are confident in their ability to provide nutrition care: A cross-sectional investigation. Public Health.

[9] Stacey D., Hopkins M., Adamo K.B., Shorr R., Prud’homme D. (2010). Knowledge translation to fitness trainers: A systematic review. Implement. Sci..

[10] Kruseman M., Miserez V., Kayser B. (2008). Knowledge about nutrition and weight loss among fitness instructors: A cross-sectional study in Geneva, Switzerland. Schweiz. Z. Fur Sportmed. Und Sporttraumatologie.

[11] Torres-McGehee T.M., Pritchett K.L., Zippel D., Minton D.M., Cellamare A., Sibilia M. (2012). Sports nutrition knowledge among collegiate athletes, coaches, athletic trainers, and strength and conditioning specialists. J. Athl. Train..

[12] Weissman J., Magnus M., Niyonsenga T., Sattlethight A.R. (2013). Sports nutrition knowledge and practices of personal trainers. J. Community Med. Health Educ..

[13] McKean M.R., Slater G., Oprescu F., Burkett B.J. (2015). Do the nutrition qualifications and professional practices of registered exercise professionals align?. Int. J. Sport Nutr. Exerc. Metab..

[14] Barnes K., Ball L., Desbrow B. (2016). Promotion of nutrition care by Australian fitness businesses: A website analysis. Public Health.

[15] Barnes K., Ball L., Desbrow B. (2017). Personal trainer perceptions of providing nutrition care to clients: A qualitative exploration. Int. J. Sport Nutr. Exerc. Metab..

[16] Feldvari K., Balog K.P., Tanacković S.F., Kurbanoğlu S.E.A. (2018). Workplace Information Literacy of Croatian Fitness and Conditioning Personal Trainers. Communications in Computer and Information Science, Proceedings of the European Conference on Information Literacy, Oulu, Finland, 24–27 September 2018.

[17] McKean M., Mitchell L., O’Connor H., Prvan T., Slater G. (2019). Are exercise professionals fit to provide nutrition advice? An evaluation of general nutrition knowledge. J. Sci. Med. Sport.

[18] Tanacković S.F., Kurbanoğlu S.E.A. (2019). Workplace Information Literacy of Croatian Fitness and Conditioning Personal Trainers. Communications in Computer and Information Science, Proceedings of the Information Literacy in Everyday Life: 6th European Conference, ECIL 2018, Oulu, Finland, 24–27 September 2018.

[19] Bianco A., Mammina C., Paoli A., Bellafiore M., Battaglia G., Caramazza G., Palma A., Jemni M. (2011). Protein supplementation in strength and conditioning adepts: Knowledge, dietary behavior and practice in Palermo, Italy. J. Int. Soc. Sports Nutr..

[20] Sanchez C., Morange P., Canault M., Tanguy S., Faille D., Dutour A., Grino M., Alessi M.-C. (2011). TOPIC 16-Rehabilitation, sport, cardiovascular prevention, obesity. Arch. Cardiovasc. Dis. Suppl..

[21] Saeedi P., Nasir M.T.M., Hazizi A.S., Vafa M.R., Foroushani A.R. (2013). Nutritional supplement use among fitness club participants in Tehran, Iran. Appetite.

[22] Gómez-Luciano C.A., Vriesekoop F., Urbano B. (2019). Towards food security of alternative dietary proteins: A comparison between Spain and the Dominican Republic. Amfiteatru Econ..

[23] Lukács A., Horváth E., Máté Z., Szabó A., Virág K., Papp M., Sándor J., Ádány R., Paulik E. (2019). Abdominal obesity increases metabolic risk factors in non-obese adults: A Hungarian cross-sectional study. BMC Public Health.

[24] Bartha É.J., Bába É.B. (2019). What type of professionals are worth being hired for fitness clubs? Study made among fitness trainers and fitness consumers. Int. Rev. Appl. Sci. Eng..

[25] Holmes C.A. (2016). Some implications of postmodernism for nursing theory, research, and practice. Can. J. Nurs. Res. Arch..

[26] Rumbold J.M.M., Pierscionek B. (2017). The effect of the general data protection regulation on medical research. J. Med. Internet Res..

[27] Szűcs Z. (2016). Okostányér–új táplálkozási ajánlás a hazai felnőtt lakosság számára. Egészségfejlesztés.

[28] Trakman G.L., Brown F., Forsyth A., Belski R. (2019). Modifications to the nutrition for sport knowledge questionnaire (NSQK) and abridged nutrition for sport knowledge questionnaire (ANSKQ). J. Int. Soc. Sports Nutr..

[29] Trakman G.L., Forsyth A., Hoye R., Belski R. (2017). The nutrition for sport knowledge questionnaire (NSKQ): Development and validation using classical test theory and Rasch analysis. J. Int. Soc. Sports Nutr..

[30] Tarján R., Bouquet D., Soós A., Walthier J. (1974). Nutritional Surveys in Hungary. Assessment of Nutritional Status and Food Consumption Surveys.

[31] Manore M.M., Hand R.K., Liguori G., Bayles M., Dolins K., Steinmuller P., Cotton R., Smith-Edge M. (2017). Knowledge and beliefs that promote or hinder collaboration among registered dietitian nutritionists and certified exercise professionals—Results of a survey. J. Acad. Nutr. Diet..

[32] Skopinceva J. (2017). Assessing the Nutrition Related Knowledge, Attitudes, and Beliefs of Fitness Professionals. Adv. Obes. Weight Manag. Control.

[33] Henard G., Luca B. (2019). Teachers’ Pedagogical Skills and Provision of Quality Education: Case of United States and Hungary. J. Educ..

[34] Végh V., Nagy Z.B., Zsigmond C., Elbert G. (2017). The effect of using edmodo in biology education on students’ attitudes towards biology and ICT. Probl. Educ. 21st Century.

[35] Abbott E. (2018). How College Students Access Nutrition Information: A Study on Social Media and Health Literacy.

[36] Compagner R. (2019). Nutrition Analysis and Comparison of Popular Social Media Recipe Videos. J. Acad. Nutr. Diet..

[37] Weeks B.E., Ardèvol-Abreu A., Gil de Zúñiga H. (2017). Online influence? Social media use, opinion leadership, and political persuasion. Int. J. Public Opin. Res..

